# Patient as teacher sessions contextualize learning, enhancing knowledge, communication, and participation of pharmacy students in the United Kingdom

**DOI:** 10.3352/jeehp.2020.17.15

**Published:** 2020-05-20

**Authors:** Andrew Martin Lunn, Ann Urmston, Steven Seymour, Andrea Manfrin

**Affiliations:** School of Pharmacy and Biomedical Science, Faculty of Clinical & Biomedical University of Central Lancashire, Preston, UK; Hallym University, Korea

**Keywords:** Education, Pharmacy, Patients, Communication, Knowledge, United Kingdom

## Abstract

**Purpose:**

This study aimed to evaluate the impact of Patient as teacher (PAT) sessions on the knowledge, communication skills, and participation of pharmacy students in the United Kingdom.

**Methods:**

During the academic year 2019–2020, year 1 and 2 pharmacy students at the University of Central Lancashire were invited to complete a questionnaire following PAT sessions. Data were analyzed by means of descriptive statistics, including mean and standard deviation for: continuous variables and reliability analysis. Pearson’s chi-square or Fisher exact test, odds ratio, and phi were used for analyzing dichotomous variables. Thematic analysis was used for free text comments.

**Results:**

Sixty eight of 228 students participated (response rate of 29.8%). No statistical difference was found between gender (P=0.090); a statistically significant difference was found between year (P=0.008). Cronbach’s α (0.809) confirmed a good internal consistency. Ninety-seven percent of the students learned a lot, and 85.3% appreciated and valued the PAT sessions; 89.7% wanted more sessions. Ninety-two point seven percent perceived the sessions to contextualize their learning. Five questions were dichotomized by grouping the responses into negative and positive; 90.3% of responses were positive and did not show statistically significant differences in gender and year of study. Overall students’ free text comments were positive, but active listening and consultation appeared in the positive and negative domains, highlighting the need for more student engagement.

**Conclusion:**

PAT sessions had a positive impact on students’ knowledge, communication skills and participation, and contextualized learning. They provide a valuable contribution to the pharmacy students’ experience in the United Kingdom.

## Introduction

### Background/rationale

The use of patients in healthcare education is well established in an acute setting; however, Patients as Teachers (PAT) in a classroom only started in the 1960s [1,2]. The level of patient involvement in the classroom has since been increasing and now varies between being used for testimony, all the way to leading sessions where they can tell their story, stimulate reflection, and help students to problem solve [3]. Pharmacy education has traditionally been science-based, but is now more clinically driven by patient facing roles, as such the inclusion of the real-world context to the curriculum is of increasing importance. Increased classroom involvement of the patient as an “expert by experience” helps to address issues in textbook teaching of chronic illness and discrepancies between theory and real-life [1,4]. PAT sessions integrate students’ learning by contextualizing theory with real patients, a requirement for the training of pharmacy students in the United Kingdom [5]. The benefits of using PAT are well documented and typically show an increase in learner satisfaction, perceived relevance of learning, and communication skills [6]. PAT sessions also provide a safe environment to practice being a healthcare professional [7]. Feedback from patients is overwhelmingly positive, feeling that they belong in the students’ education, enjoying giving back to the community, and reporting benefits to their self-esteem and personal health. Patient concerns focus on anxiety about communicating their story, engaging, and educating the students [7]. These concerns are addressed with adequate patient selection and training; if done well, the patients become “colleagues in teaching” [4,6]. PAT sessions are utilized in the training of healthcare professionals and have been extensively reviewed, showing good evidence of short-term benefit to learning and satisfaction and facilitating deeper learning. They allow the application of knowledge by “showing how” and “doing”, rather than by a simple factual recall according to Miller’s pyramid [4,7,8]. However, the literature has focused on the training of physicians and nurses, with the impact of such sessions on pharmacy students less thoroughly explored [1].

The PAT sessions delivered at the University of Central Lancashire (UCLan) cover 10 areas: cardiovascular, central nervous system, endocrine, gastrointestinal, genitourinary, hearing, musculoskeletal, respiratory, sight, and skin. During the sessions, students spend time with different patients, practicing their clinical and communication skills, with elements that are teacher-led, patient-led, and jointly led by patients and teachers, and discussions. Similar PAT sessions are utilized at many UK pharmacy schools including the University of Sussex, Medway School of Pharmacy, and University College London.

### Objectives

The study aimed to evaluate the impact of PAT sessions on knowledge, communication skills, and participation of pharmacy students in the United Kingdom.

The key research questions of the study were as follows: First, do PAT sessions contextualize learning? Second, do PAT sessions have an impact on students’ knowledge, communication, and participation?

## Methods

### Ethics statement

The study was conducted in accordance with the Helsinki Declaration of 1975 as revised in 2008 and received ethical approval from the Health Ethics Review Panel of the University of Central Lancashire on January 6th, 2020 (approval no., HEALTH 0029). Informed consent was obtained from all individual participants included in the study. All data were handled following the requirements of the Data Protection Act (2018) and/or the General Data Protection Regulation 2016 according to European Union law; therefore, data were anonymized and stripped of any identifiable references to the participants.

### Study design

This was a single institute survey-based study.

### Population

In this study, first and second-year pharmacy students were invited to participate. These years were chosen as the sessions were comparable in delivery, allowing a combination of data. The 15 PAT sessions were delivered to first- and second-year students in term 1 (September–December 2019) and term 2 (January–April 2020) and are summarized in Table 1. Ethics approval was received at the beginning of term 2; therefore, the recruitment and the study were conducted in term 2 during the 2019–20 academic year.

### Commensus at the University of Central Lancashire

Commensus (Community Engagement, Service User Support) is a service user, carer, patient, and public group based at the UCLan, which was set up in 2004 [9]. The group currently works to embed authentic public voices and experiences in the teaching and learning of current and future professionals from individual perspectives [10]. These volunteers are recruited through these organizations, by staff and students in practice, and from attendance at public engagement events, online marketing, and word of mouth. The volunteers provide their time freely and are only paid theirs out of pocket expenses. They are supported by dedicated and experienced facilitators who recruit, train, and support the volunteers, and offer guidance and advice to staff within the schools around this area.

### Measurement

The research instrument was a questionnaire previously used by Costello and Horne [6] in 2001 aiming at rating student’s satisfaction, perception of learning, and level of involvement. The questionnaire had 7 question items, which was a mix between a 5-point Likert scale and binary agree/disagree options. The questionnaire also gathered students’ comments on the PAT sessions. For our research, we added a demographic section (5 items) and four additional 5-point Likert scale items previously used in another project aimed at assessing the impact of PAT sessions on student’s contextualization of learning, communication, confidence, and enthusiasm [11]. Permission to use the questionnaire was received from the original publishers Elsevier. Following informed consent, students were invited to fill out an online questionnaire delivered through a web platform called Qualtrics available from https://www.qualtrics.com (Full questionnaire available in Dataset 1).

### Study power

A post hoc power calculation was conducted using G*Power ver. 3.1.9.4 (Heinrich-Heine-Universität Düsseldorf, Düsseldorf, Germany; http://www.gpower.hhu.de/) [12], and Pearson’s chi-square was the statistical test used. There was a sample size of 68 students, the effect size (Cohen d) of 0.5, and an alpha error of 0.05, and the calculated power was 91% with a critical chi-square of 11.07 and 5 degrees of freedom.

### Data analysis

Descriptive statistics were used for presenting the table using categorical variables. Data were presented as a range, mean, and standard deviation as suggested by Norman [13].

### Reliability analysis

Cronbach’s α reliability coefficient normally ranges between 0 and 1. The closer Cronbach’s α coefficient is to 1.0, the greater the internal consistency of the items in the scale. Field suggested that the value of alpha depends on the number of items on the scale [14]. For this reason, as the number of items on the scale increases, alpha increases too. If the number of items on the scale is less than 10, alpha should be ≥0.5. There is a formula for the calculation of alpha, α=rk/{1+(k–1) r} where k is the number of items considered and r is the mean of the inter-item correlations the size of alpha is determined by both the number of items in the scale and the mean inter-item correlations. A general rule of thumb for internal consistency suggests that alpha >0.9, excellent; >0.8, good; >0.7, acceptable; and >0.6, questionable. It is important to note that while a high value for Cronbach’s α indicates good internal consistency of the items in the scale (reliability), it does not mean that the scale is unidimensional.

### Dichotomization of the variables and measure of association

Some variables were dichotomized, polarizing the responses into negative and positive as suggested by Aires et al. [1]. “Strongly agree” and “agree” were grouped as positive, adopting a conservative approach; “unsure” was grouped with “disagree”, and “strongly disagree” as negative. The dichotomization process allowed the measurement of the odds ratio and the association between categorical variables with a binary option (2×2). We used the phi (φ) coefficient (or mean square contingency coefficient) to measure the association between two binary variables. Phi is measured similarly to Pearson’s correlation coefficient in its interpretation, represents the chi-square-based measure of association. The chi-square coefficient depends on the strength of the relationship and the sample size. Phi eliminates sample size by dividing chi-square by n, the sample size, and taking the square root. The values of the phi coefficient range between -1 (negative association) and +1 (positive association).

### Thematic analysis

The text responses to the questions were examined, and preliminary codes were given; the search for patterns was developed, and a mind map constructed. Common themes were identified and grouped. Participants’ comments were grouped according to the themes.

The analyses were conducted using IBM SPSS ver. 26.0 (IBM Corp., Armonk, NY, USA) and Microsoft Excel ver. 2016 (Microsoft Corp., Redmond, WA, USA). NVivo ver. 12 (QSR International, Melbourne, Australia) was used for the generation of the mind-map and thematic analysis. A P-value <0.05 was considered to indicate statistical significance.

## Results

### Participants’ demographic characteristics

The total number of students in years 1 and 2 was 228 (year 1=129; year 2=99). The number of students who participated in the study was 68, giving a response rate of 29.8%; 60.3% were female (P=0.090), and 66.2% were in the first year and 33.8% in the second (P<0.008). The percentage of female students in year 1 was 55.6 and in year two 69.6; the difference was not statistically significant (P=0.305) (Table 2).

### Internal consistency

The reliability of the questionnaire was assessed using Cronbach’s α which measures the internal consistency of the scale, and therefore, how closely related a set of items (questions) are as a group. The questions not related to the PAT activities, such as demographic, were excluded from the analysis. Cronbach’s α was assessed on 9 items; the value obtained (0.809) confirming a good internal consistency (Tables 3, 4).

### Appreciation of Patients as Teachers sessions

Students were asked to rate their appreciation of the PAT sessions using a scale from 1 (least satisfactory) to 5 (most satisfactory). Over 38% (38.2%) rated 5, 4 (47.1%), 3 (10.3%), and 2 (4.4%). Students suggested that the most worthwhile aspects of PAT were the joint elements run by both teachers and patients (55.9%), followed by patient-led (17.6%), discussion (16.2%), and teacher-led (10.3%).

Student responses to statements: all the statements presented in Table 5 were very positive, suggesting that students learned from the sessions; most of the students (97.0%) learned a lot, an adequate amount or a great deal; and only 3.0% learned very little. The patient involvement helped the students to acquire a greater understanding of patient’s problems, and 89.7% would like to see more PAT sessions. The PAT sessions contributed to contextualize students’ learning, communication skills, confidence, and enthusiasm (participation) in 92.7% of the sample (30.9% strongly agree; 61.8% agree) (The full list of pros and cons from students comments can be found in Supplement 1).

### Dichotomized options

Five questions were dichotomized for grouping the responses into positive and negative. The results presented in Tables 6 and 7 did not show statistically significant differences between gender and year of study. Nevertheless, both tables are showing a robust positive appreciation of the PAT sessions.

### Thematic analysis

**Fig. 1.**
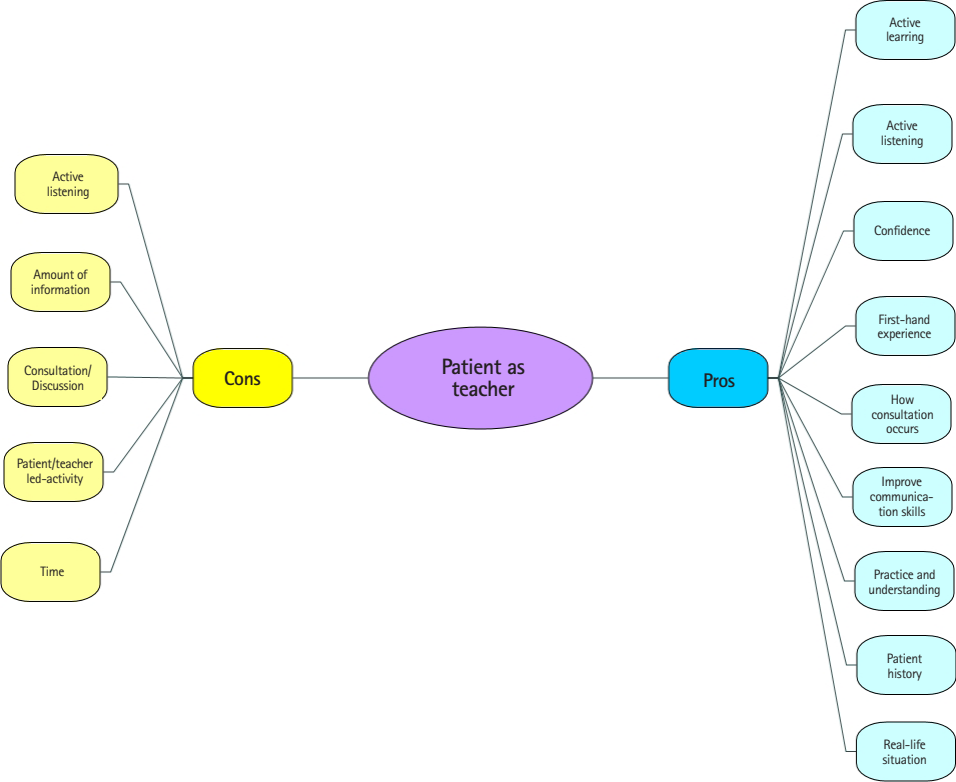
Patient as teacher, student comments mind map.

Students were invited to write comments regarding the PAT sessions. The PAT mind map is summarising the pros and cons perceived by students during the sessions (Fig. 1), which have been grouped into themes and described in detail in Supplement 1.

## Discussion

The student response rate was 29.8%, with 60.3% of respondents being female. Dichotomization of data showed no statistically significant difference in response between gender and year, suggesting that PAT sessions were perceived equally by male and female, and first- and second-year students. Eighty-five point three percent of the students rated their appreciation of the sessions as 4 or 5 (out of 5), indicating that students appreciate PAT sessions and recognized their value. These results were re-enforced by the much lower number of comments left in the negative feedback section (7, with 2 of these being positive), compared to 41 positive statements. Aires et al. [1] conducted a study where PAT sessions were involved in training general practitioners in France; the results confirmed the appreciation of these sessions, which helped general practitioners to develop competencies by providing patient-specific content.

When asked to choose which part of the session was most worthwhile, students showed a clear preference for components led jointly by patients and teachers (55.9%), compared to solely patient-led (17.6%). This shows a difference to previous studies such as that by Towle et al. [2], which suggested the most worthwhile components of PAT sessions were those led by the patient. The study of Towle et al. [2] predominantly included nursing, occupational therapy, and medical students, which focused on PAT sessions led independently by patients, with students having multiple prolonged sessions with one patient. Whereas this research focuses on a more structured environment, with multiple shorter encounters with different patients and exclusively pharmacy students. Such differences might show the importance of the PAT session structure and the level of teacher involvement in how students perceive sessions and the relative differences in perception between students of different professions. The authors of an integrative literature review on the use of standardized patients in pharmacy education identified 4 themes; (1) student satisfaction, (2) effectiveness to confer knowledge, (3) skills and interprofessional practices, and (4) the use of PAT in assessment and the cost of the educational intervention. Themes 1, 2, and 3 were identified in this study too [15]. Student preference of the combined patient-teacher components was re-enforced by the thematic analysis. In contrast, the elements led by patients or teachers alone received negative feedback citing the amount of information presented and time spent with each patient as issues.

When students were asked to comment on the positive aspects of the PAT sessions, common themes emerged around confidence, communication, and contextualization (integration) of learning. Combined with the questionnaire responses, students perceive the PAT sessions to: contribute greatly to learning; help understand the patient perspective; take learning beyond the textbook; and improve the skills and have confidence in communicating with patients.

These results contribute to higher student satisfaction, with 89.9% of respondents wanting more PAT sessions. Furthermore, 92.7% of respondents also agreed that the sessions contextualized their learning. This finding suggests that using patients as teachers is an effective way to integrate curriculum teaching into practice in a pharmacy course, as required by the General Pharmaceutical Council [5].

When looking at the themes arising in the positive and negative comments (Fig. 1), active listening and consultation can be seen to appear on both sides, highlighting the importance of incorporating a range of activities into sessions to engage all students.

### Strengths and limitations

Data for this study was collected exclusively from years 1 and 2 pharmacy students over one term with the same patients for each session. This allows for a greater consistency that would not be possible over a longer time or with variation in patients and teachers; this does, however, mean that the data are less generalizable. A significant limitation of this study is the small sample size which means that it is difficult to draw strong conclusions.

### Conclusion

The study has shown that PAT sessions are seen as valuable learning tools by pharmacy students, who perceived an improvement in their communication skills and confidence. Students also value them as a way to contextualize learning, taking it out of the classroom and integrating knowledge into practice.

## Figures and Tables

**Table 1.** Details of PAT sessions studied for pharmacy students at the University of Central Lancashire

	Details
Structure of PAT sessions	
Year 1	Session 1: Students are introduced to patients through as a meet and greet, and different styles of questioning and how to overcome barriers are taught.
	Session 2: The students carry out activities with the patients regarding active listening, questioning, and consultations.
	Session 3: Students participate in a Q&A session around medicine storage at home, medicine compliance, and clinical trials.
Year 2	Students cover 8 body systems and have one PAT session for each body system throughout the year. These sessions involve a patient discussing a condition linked to the relevant body conditions as single morbidities.
Delivery of PAT sessions	All PAT sessions are delivered in a similar format. The students are set pre-work, for example to research and think about the types of questions they would ask a patient with the condition that will be covered. In the classroom, students are split into groups (typically 4–6 students) and work with a patient for 20 minutes. Depending on what year group/session they are on, the students are set themes to cover and gain further information about from patients. The student groups then rotate, allowing the students to meet different patients with different experiences. Sessions vary in the patients present depending on topics covered and availability; however, all patients receive the same training.
How often PAT sessions are delivered	In year 1, students have 3 sessions, one in the first semester and 2 in the second semester. In year 2, students have 10 sessions, 5 in each semester. All sessions are around 2 hours in length.

PAT, Patient as teacher.

**Table 2.** Participants’ demographic characteristics

Characteristic	No. (%)
Gender	
Female	41 (60.3)
Male	27 (39.7)
Age group (yr)	
>20	25 (36.8)
19	21 (30.8)
20	11 (16.2)
18	11 (16.2)
Ethnic group	
Asian/Asian British	50 (73.6)
White	9 (13.2)
Black/African/Caribbean/Black British	5 (7.4)
Chinese or other ethnic groups	2 (2.9)
Mixed/multiple ethnic groups	2 (2.9)
Year	
First	45 (66.2)
Second	23 (33.8)

**Table 3.** Reliability analysis: statistics for scale

Variable	Value
Statistics for scale	
No. of items	9
Mean±standard deviation	38.93±4.053
Variance	16.427
Item means	
Mean	4.325
Min–max (range)	3.176–6.176 (3.000)
Min/max	1.944
Variance	0.628
Item variances	
Mean	0.512
Min–max (range)	0.297–1.133 (0.836)
Min/max	3.817
Variance	0.066

**Table 4.** Reliability analysis for 9 items

Item total statistics	Scale mean if item deleted	Scale variance if item deleted	Corrected item-total correlation	Squared multiple correlation	Cronbach’s α if item deleted
On a scale of 1 (least satisfactory) to 5 (most satisfactory) how would you rate the teaching session?	34.74	12.078	0.671	0.543	0.767
Which aspect of the session did you find the most worthwhile?	35.75	12.280	0.404	0.255	0.819
How much did you learn from the session about the care of the patient?	35.04	12.640	0.612	0.464	0.776
The involvement of a patient in the session helped me to gain a greater understanding of the patients’ problems	34.47	13.536	0.593	0.437	0.783
Would you like to see more of this type of session?	32.75	17.175	-0.231	0.168	0.856
Learning from expert patients helped to contextualize my learning	34.71	12.808	0.730	0.668	0.766
Learning from expert patients helped to improve my communication & consultation skills	34.53	13.238	0.585	0.540	0.781
My confidence when talking to patients was improved by the patient encounter	34.75	12.907	0.738	0.597	0.766
The expert patient generated interest and enthusiasm during the session	34.68	12.939	0.596	0.392	0.779

Reliability coefficient for 9 items: α=0.809; standardized item α=0.813.

**Table 5.** Student responses to statements

Statement	No. (%)
How much did you learn from the session about the care of the patient?	
A lot	36 (52.9)
Adequate amount	17 (25.0)
A great deal	13 (19.1)
Very little	2 (3.0)
The involvement of a patient in the session helped me to gain a greater understanding of the patients’ problems	
Strongly agree	34 (50.0)
Agree	31 (45.6)
Unsure	3 (4.4)
Would you like to see more of this type of session	
Yes	61 (89.7)
Not sure	5 (7.4)
No	2 (2.9)
Learning from expert patients helped to contextualize my learning	
Agree	42 (61.8)
Strongly agree	21 (30.9)
Unsure	4 (5.9)
Disagree	1 (1.4)
Learning from expert patients helped to improve my communication & consultation skills	
Agree	32 (47.1)
Strongly agree	32 (47.1)
Unsure	3 (4.4)
Disagree	1 (1.4)
My confidence when talking to patients was improved by the patient encounter	
Agree	42 (61.8)
Strongly agree	19 (27.9)
Unsure	7 (10.3)
The expert patient generated interest and enthusiasm during the session	
Agree	34 (50.0)
Strongly agree	26 (38.2)
Unsure	7 (10.3)
Disagree	1 (1.5)

**Table 6.** Binary options using gender as a dichotomous variable

Statement	No. of male (%)	No. of female (%)	Odds ratio (95% confidence interval)	Strength of association (phi)	P-value
The involvement of a patient in the session helped me to gain a greater understanding of the patients’ problems			2.879 (0.627–13.223)	0.170	0.250
Agree	22 (81.5)	38 (92.7)			
Disagree	5 (18.5)	3 (7.3)			
Learning from expert patients helped to contextualize my learning			0.356 (0.038–3.369)	-0.113	0.641
Agree	26 (96.3)	37 (90.2)			
Disagree	1 (3.7)	4 (9.8)			
Learning from expert patients helped to improve my communication & consultation skills			1.560 (0.206–11.798)	0.053	1.000
Agree	25 (92.6)	39 (95.1)			
Disagree	2 (7.4)	2 (4.9)			
My confidence when talking to patients was improved by the patient encounter			0.576 (0.103–3.208)	-0.077	0.694
Agree	25 (92.6)	36 (87.8)			
Disagree	2 (7.4)	5 (12.2)			
The expert patient generated interest and enthusiasm during the session			2.879 (0.627–13.223)	0.170	0.250
Agree	22 (81.5)	38 (92.7)			
Disagree	5 (18.5)	3 (7.3)			

P-values are expressed as Pearson’s chi-square (X^2^) or Fisher exact test; statistically significant P<0.005. Phi shows the strengths of the association between 2 variables (-1≤ phi ≤+1). Agree includes strongly agree and agree. Disagree includes strongly disagree, disagree, and unsure.

**Table 7.** Binary options using the year as a dichotomous variable

Statement	No. of year 1 (%)	No. of year 2 (%)	Odds ratio (95% confidence interval)	Strength of association (phi)	P-value
The involvement of a patient in the session helped me to gain a greater understanding of the patients’ problems			0.833 (0.181–3.843)	-0.280	1.000
Agree	40 (88.9)	20 (87.0)			
Disagree	5 (11.1)	3 (13.0)			
Learning from expert patients helped to contextualize my learning			0.310 (0.048–2.004)	-0.156	0.327
Agree	43 (95.6)	20 (87.0)			
Disagree	2 (4.4)	3 (13.0)			
Learning from expert patients helped to improve my communication & consultation skills			0.488 (0.064–3.712)	-0.085	0.599
Agree	43 (95.6)	21 (91.3)			
Disagree	2 (4.4)	2 (8.7)			
My confidence when talking to patients was improved by the patient encounter			0.065 (0.133–3.188)	-0.065	0.681
Agree	41 (91.1)	20 (87.0)			
Disagree	4 (8.9)	3 (13.0)			
The expert patient generated interest and enthusiasm during the session			0.833 (0.181–3.843)	-0.028	1.000
Agree	40 (88.9)	20 (87.0)			
Disagree	5 (11.1)	3 (13.0)			

P-values are expressed as Pearson’s chi-square (X^2^) or Fisher exact test; statistically significant P<0.005. Phi shows the strengths of the association between 2 variables (-1≤ phi ≤+1). Agree includes strongly agree and agree. Disagree includes strongly disagree, disagree, and unsure.

## Footnotes

**Data availability** Data files are available from Harvard Dataverse: https://doi.org/10.7910/DVN/6H1ZR6 Dataset 1. Data file & data dictionary (Excel format 2016).

## Supplementary materials

Data files are available from Harvard Dataverse: https://doi.org/10.7910/DVN/6H1ZR6

Supplement 1. Pros and cons of PAT sessions according to students’ comments.

Supplement 2. Audio recording of the abstract.
