# Supplementary material for: Patient as teacher sessions contextualize learning, enhancing knowledge, communication, and participation of pharmacy students in the United Kingdom
Source: J Educ Eval Health Prof. 2020 May 20;17:15. doi: 10.3352/jeehp.2020.17.15 (PMC7344118; doi:10.3352/jeehp.2020.17.15)
Supplement: Supplementary file 2 — Supplement 1. Pros and cons of PAT sessions according to students’ comments. [file jeehp-17-15-suppl.docx]

**Suppl. 1. Pros and cons of PAT sessions according to students’ comments**

The cons domain grouped the following themes: 1) time, 2) active listening, 3) consultation and discussion, 4) patient /teacher and led activities, 5) amount of information.

**Cons (230)**

Twenty-seven students provided comments regarding the drawbacks of the PAT sessions, and some of them are summarised in this section.

1. **Time**

One student said

"*A little too long with each patient- no more questions to ask/ aspects to discuss so can be a little awkward*"

[Student 17, year 2]

1. **Active listening**

Seven first-year students said that active listening was the aspect that they find the least worthwhile.

1. **Consultation and discussion**

The consultation and questioning time was also identified as the least worthwhile.

"*Questioning can tend to go off-topic*"

[Student 55, year 1]

1. **Patient/teacher-led activity**

Two students suggested that the teacher-led activity was the least worthwhile, and two the patient-led.

1. **Amount of information**

Some students identified the teacher-led activities as least worthwhile as they had too much information.

"*Teacher-led as I felt as though too much information was thrown at me during the lecture. It didn't really sink in. I have to go over it a number of times to understand it fully*".

[Student 46, year 1]

**Positive comments in the cons section**

Other students replied using positive statements such as

"*None all aspects were great*".

[Student 58 year 2]

"*I think there aren't any negative aspects as the session was valuable because it gave me more experience and confidence*".

[Student 14, year 1]

"*Not applicable, I really enjoyed the session*".

[Student 53 year 1]

**Pros (425)**

Forty-one students provided text comments regarding the advantages of PAT sessions, and the themes and some comments are listed in this section. The pros domain grouped the following themes: 1) active learning, 2) active listening, 3) confidence, 4) first-hand experience, 5) how consultation occurs, 6) improve communication skills, 7) practice and understanding, 8) patient history, 9) real situation.

1. **Active learning**

"*I am an active learner and learn more from activities than from lectures. So, discussing and asking questions with patients I feel is a more effective way of getting some information*"

[Student 58, year 2]

1. **Active listening**

"*Listen actively to the real Patient and understand their needs and what matters to them. Ask question and listen carefully to the responses, check the Patient has understood the information they have been given*".

[Student 14, year 1]

1. **Confidence**

"*Confidence with talking to patents*"

[Student 3, year 1]

"*Confidence In experiencing patient; pharmacist interactions*"

[Student 4, year 1]

"*Gave me more self-confidence when speaking with patients*"

[Student 37, year 1]

1. **First-hand experience**

"*Provides a first-hand example of how these conditions can affect a person day to day life*"

[Student 8, year 2]

"*Helps us get a first-hand opinion of the diseases we are studying and helps us better understand the symptoms patients experience, as well as the medicine they take and if it is actually working for them*"

[Student 43, year 2]

1. **How consultation occurs**

*"I understood about the different aspects I should look out for during a consultation"*

[Student 25, year 1]

1. **Improve communication skills**

"*It would enable me to develop and enhance my communication skills further as I would become more confident in speaking and expressing my feelings*"

[Student 47, year 1]

1. **Practice and understanding**

"*It allows us to ask more question to patients and see their expression and listen to their stories. It helps me understand more about their condition and lifestyle too. Also, it helps to practice the type of questions that should be asked and how they should be, some patients may be sensitive to certain question, therefore these sessions help tackle this*".

[Student 46, year 2]

1. **Patient history**

"*Understanding their story*"

[Student 26, year 1]

1. **Real situation**

"*Get real-life experience and have more confidence*"

[Student 27, year 2]

"*Get to learn about real-life scenarios and apply what we have been learning to a direct situation*"

[Student 42, year 2]

"*Personally, it was like a real experience and helped me to understand how it will be in the real-life*"

[Student 63, year 1]
